# Uric acid levels among Tunisian patients with bipolar disorder during different phases of illness

**DOI:** 10.1192/j.eurpsy.2022.1045

**Published:** 2022-09-01

**Authors:** D. Falfel, G. Hamdi, H. Ben Ammar, R. Ridha

**Affiliations:** Razi Hospital, Psychiatry Ward, Manouba, Tunisia

**Keywords:** Depression, uric acid, manic episod, bipolar disorder

## Abstract

**Introduction:**

Bipolar disorder is a recurrent chronic disorder characterised by fluctuation of mood state. Recent studies focused on the involvement of adenosine and the purinergic system in the pathophysiology of bipolar disorder.

**Objectives:**

We aimed to investigate the difference in (SUA) levels between different phases of relapse and remission period.

**Methods:**

For this aim a prospective study was conducted during six months at Razi psychiatric Hospital in Tunisia with patients diagnosed with BD The socio-demographic, clinical data were gathered from patient and a psychometric assessment using YMRS and Beck scale was employed. Uric acid level was studied during relapse and remission period.

**Results:**

Among 30 consentent patients included in the study : 65.7% were women, The age of the participants varied between 22 and 65 years old. Uric acid level at the relapse varied between 328 and 499 mmol/L and level of controlled value at the remission period which is eight weeks under treatment varied between 137 and 307 mmol/L. Patients under antipsychotic treatment were 55.9% the other were under lithium or mood stabilizer. There is no significant difference between patients with bipolar disorder type I or II neither for the molecule chosen for treatment (p<0.05).

**Conclusions:**

Bipolar disorder is a chronic psychiatric disease which needs to be regulary controlled. Uric acid levels were higher in manic or depressive phases as compared with euthymia phase. Uric acid could be used as a trait marker in bipolar disorder and help psychiatrist to monitor patients and to adjust treatment in order to avoid relapsing.

**Disclosure:**

No significant relationships.

